# Input and output pathways determining potassium budgets in two paddy fields subjected to countermeasures against radiocesium in Fukushima, Japan

**DOI:** 10.1371/journal.pone.0232139

**Published:** 2020-04-24

**Authors:** Tatsuhiro Nishikiori, Tomijiro Kubota, Susumu Miyazu, Naoki Harada, Natsuki Yoshikawa, Hideshi Fujiwara, Takashi Saito

**Affiliations:** 1 Agricultural Radiation Research Center, Tohoku Agricultural Research Center, National Agriculture and Food Research Organization, Fukushima-shi, Fukushima, Japan; 2 Institute for Rural Engineering, National Agriculture and Food Research Organization, Tsukuba, Ibaraki, Japan; 3 Faculty of Agriculture, Niigata University, Nishi-ku, Niigata-shi, Niigata, Japan; 4 Institute for Agro-Environmental Science, National Agriculture and Food Research Organization, Tsukuba, Ibaraki, Japan; 5 Hama Agricultural Regeneration Research Centre, Fukushima Agricultural Technology Centre, Minamisoma, Fukushima, Japan; Universidade de Santiago de Compostela, SPAIN

## Abstract

Countermeasures to reduce radiocesium (^134^Cs and ^137^Cs) uptake by crops have been implemented in farmlands affected by the Fukushima nuclear accident in 2011. A widely practiced countermeasure is the application of potassium (K). Long-term soil K maintenance is a key issue due to the long physical half-life of ^137^Cs (30 years). Information on input and output pathways determining plant-available K budgets can provide a base for the development of maintenance strategies. Therefore, in this study we evaluated these pathways in paddy fields subjected to K fertilization as a countermeasure. We selected two fields with different soil textures and drainage conditions and quantified input and output via fertilization, irrigation, precipitation, straw return to soil, plant harvesting, surface runoff, and percolation during the cropping period in 2018. The major input pathways were fertilization, straw return, and irrigation due to a large inflow volume with spill-over irrigation. The major output pathways consisted of plant harvesting, surface runoff, and percolation. However, 85% of K in harvested plants was brought back by straw return; in practice, harvesting was a minor pathway. The K budgets during the study period were negative (−20 and −289 kg ha^−1^) and especially severe in clay loam soil with high output via percolation. This could probably be attributed to the low cation exchange capacity and high permeability from the low total C and clay contents. Losses via surface runoff stemmed from excessive irrigation volumes in both fields. Around 70% of the total K output via surface runoff and percolation was discharged before mid-summer drainage. Accordingly, controlling the irrigation volume during this period in addition to increasing cation exchange capacity and decreasing permeability may improve the negative budgets.

## Introduction

The Fukushima Daiichi Nuclear Power Plant accident following the Great East Japan Earthquake on March 11, 2011 resulted in the emission of a large amount of radionuclides (^131^I, ^134^Cs, and ^137^Cs) into the atmosphere, thereby contaminating a wide area of eastern Japan. ^131^I has a short physical half-life (8 days) and does not remain in the environment for years. ^134^Cs and ^137^Cs, however, have long half-lives (2 and 30 years, respectively). Numerous studies have shown that the removal of radiocesium by water discharge from watersheds [e.g., 1–4] is limited due to its strong adsorption by clay minerals in soil [5], leading to the potential for prolonged contamination.

Most radiocesium has not been discharged from agricultural areas at the field scale [6–9]; accordingly, countermeasures against the contamination of agricultural products have been implemented. Current guidelines [10] recommend maintaining the soil exchangeable K content to reduce radiocesium uptake by crops. This is based on the competition between K and Cs for uptake by plants. Although the target K content in cultivated rice is >0.20 cmol K kg^−1^ in Fukushima under conventional fertilization [11], the content is increased to greater than 0.53 cmol K kg^−1^ by fertilization with potassium chloride and manure and by straw return to soil before conventional fertilization. Although topsoil removal at a depth of 5 cm has been performed in highly contaminated farmlands (>5000 Bq kg^−1^ in soil) as a countermeasure [12], it is difficult to remove radiocesium that migrates to the lower layer [8,13]. Radiocesium is occasionally retained at thousands of becquerels per kilogram even after decontamination [14]. Thus, soil K maintenance is an important long-term issue for the sustainable production of safe crops.

The maintenance of K, a major essential element for crops, is critical for meeting the growing food demand as the global population increases. Because information on K input and output pathways determining the K budget can inform maintenance strategies, extensive research has evaluated these pathways and budgets under various crops and cultivation systems. Various pathways are involved in input (fertilization, irrigation, precipitation, capillary rise, and straw return to soil) and output (plant harvesting, surface runoff, and percolation) in irrigated rice cropping systems. Naturally, the primary input pathway is fertilization, whereas irrigation provides a non-negligible quantity of K, accounting for 5%–36% of the total input [15–19]. Straw return is another important pathway [20–22].

Plant harvesting is a major output pathway. However, output via drainage containing surface runoff or percolation is typically not included in the budget calculation, unlike in N budget studies, with a few exceptions [15,16]. The output is expected to vary depending on K adsorption in soil and drainage conditions [23]. Additional observations of output pathways are needed under different conditions to clarify their effects on the budget.

Our final goal is to develop an appropriate K maintenance plan to control long-term radiocesium uptake in paddy fields affected by the Fukushima nuclear accident. In the present study, we aimed to determine the major K pathways contributing to the plant-available K budgets of paddy fields with countermeasures against radiocesium. In particular, we selected two fields with different soil textures and drainage conditions in Fukushima, quantified K input and output via fertilization, irrigation, precipitation, straw return, plant harvesting, surface runoff, and percolation, and calculated K budgets.

## Materials and methods

### Site description

Study fields were selected from evacuation zones within 10 km of the Fukushima Daiichi Nuclear Power Plant to support the resumption of farming. We obtained permission from the landowners to use the study fields. The evacuation order in the study area was lifted by 2019. The total deposition density of ^134^Cs and ^137^Cs was 1000–3000 kBq m^−2^ (decay correction date: November 5, 2011) according to an airborne monitoring survey [24]. The fields were situated on a coastal plain in Fukushima Prefecture. Field A was located 37°23'01"N and 140°58'19"E at an altitude of 72 m, and it occupied an area of 755 m^2^. Field B was located 37°30'01"N and 140°58'25"E at an altitude of 13 m, occupying an area of 400 m^2^. At the nearest meteorological weather station (AMeDAS Namie) in 2009–2018, the mean temperature was 13.0°C and the precipitation was 1500 mm [25].

Soil types were Andosols for field A and Gray Lowland soils (Gleyic Fluvisol) for field B. The fields were decontaminated by removal of plowed soil at a depth of approximately 5 cm and the addition of uncontaminated soil. The basic properties of plowed soil are presented in Table 1. Soil in field A was characterized by heavy clay and relatively high total C, N, and cation exchange capacity (CEC). Field B consisted of clay loam including gravel and had lower total C, N, and CEC but higher exchangeable and non-exchangeable K than those of field A. Soil texture was classified based on the system defined by the International Soil Science Society, revised by Yamanaka [26]. Saturated hydraulic conductivities of the plowed layer were comparable, while those of the plowsole layer were two orders of magnitude higher in field B than in field A.

**Table 1:** Basic properties of plowed soils.

Soil properties	Field A	Field B
Bulk density (kg dm^−3^)	0.90	1.43
Clay (<0.002 mm) (%)	54	16
Silt (0.002–0.02 mm) (%)	19	27
Sand (0.02–2 mm) (%)	27	54
Gravel (>2 mm) (%)	0	4
pH (H_2_O)^a^	5.5	5.9
Total C (g kg^−1^)	48	12
Total N (g kg^−1^)	3.4	1.0
Cation exchange capacity (cmol_c_ kg^−1^)^b^	20.3	9.0
Exchangeable K (cmol kg^−1^)^c^	0.38	0.74
Non-exchangeable K (cmol kg^−1^)^d^	0.91	1.09
Exchangeable K stock (kg ha^−1^)^e^	202	624
Non-exchangeable K stock (kg ha^−1^)^e^	479	918
Saturated hydraulic conductivity of plowed layer (cm sec^−1^)^f^	1.2 × 10^−5^	2.5 × 10^−5^
Saturated hydraulic conductivity of plowsole layer (cm sec^−1^)^f^	3.4 × 10^−7^	4.9 × 10^−5^

Soil samples were collected during the harvesting season in 2018.

^a^Soil: water = 1:2.5 w/v [27].

^b^Semimicro-Schollenberger method [27].

^c^1 M ammonium acetate extraction (soil: solution = 1:10 w/v).

^d^K extracted with sodium tetraphenylborate [28] minus exchangeable K.

^e^Values computed by multiplying the soil volume (bulk density × plowed soil thickness of 15 cm) and K concentrations.

^f^Falling head permeability test [27].

The general cropping system in the area is monoculture paddy fields. Rice seedlings (*Oryza sativa* L., Koshihikari) were transplanted in May and harvested in October 2018. Midsummer drainage was carried out in the middle of July for water management in paddy fields. Fertilizer application rates of N-P-K (kg ha^−1^) were 4-0-25 for field A and 82-75-149 for field B. The rates of K were set before transplanting to meet recommended guidelines for the soil exchangeable K content (>0.53 cmol K kg^−1^) [10]. The primary types of N, P, and K fertilizers were ammonium sulfate, fused magnesium phosphate, and potassium chloride, respectively. Fertilizers were applied before transplanting, except for N in field A (top-dressing was completed in early July). Harvested straw was returned to the fields immediately after harvesting. Irrigation water sources were a neighboring dam and river. The management of irrigation, transplanting, and fertilization was outsourced to farmers. The farmer at field A commuted from an area outside of the evacuation zone, and water was supplied by spill-over irrigation in field A and intermittent irrigation in field B. Observations began at the rice transplanting stage in field A (May 9 to October 2) and before pudding in field B (May 10 to October 2).

### Hydrological observation

Flow rates of irrigation and surface runoff were recorded at 10-min intervals using electromagnetic flow meters (U-K, Aichi Tokei Denki Co., Ltd., Nagoya, Japan) and Parshall flumes (2-inch type; UIZIN Co. Ltd, Tokyo, Japan). Flooding water depth was monitored at the same interval using water gauges (HTV-020KP; Hi-net, Tokyo, Japan). The mean water requirement in depth at night with no precipitation and no irrigation was used as the percolation rate. The amount of precipitation in field A was based on data from meteorological weather stations in close proximity (AMeDAS Namie and Tomioka) [25]. Precipitation in field B was observed using a rain gauge (OW-34-BP; Ota Keiki Seisakusho Co., Ltd., Tokyo, Japan). Crop evapotranspiration was quantified according to Food and Agriculture Organization guidelines [29] by multiplying reference crop evapotranspiration by the crop coefficient for rice. Data from nearby meteorological weather stations (AMeDAS Namie, Tomioka, and Onahama) [25] were used for calculations.

### Sampling and K analysis

**Fig 1:**
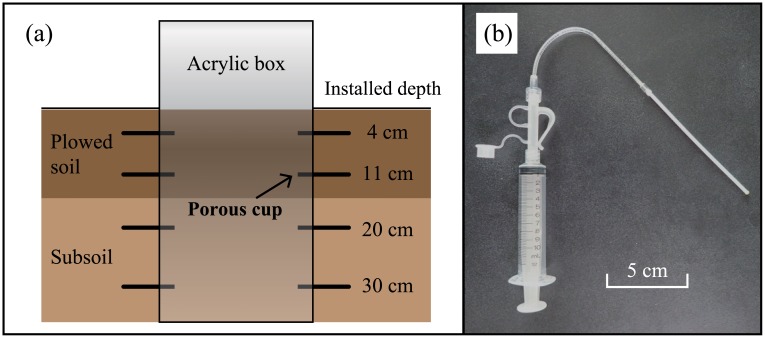
(a) Schematic diagram of soil solution sampling and (b) photograph of a sampling device consisting of a porous cup and a syringe.

Irrigation water was collected from canals every day or every week in polypropylene bottles. Ponding water near the outlet was collected in the same way and treated as surface runoff water. Precipitation was collected using a stainless-steel pan with an area of 0.5 m^2^. Precipitation sampling was started in August at field B, and cation concentrations (including K) recorded at field A were substituted for those from May to July in field B. Acrylic boxes were installed in the fields at locations away from inlets and outlets to obtain soil solutions from plowed soil and subsoil. Samples were collected from depths of 4, 11, 20, and 30 cm in triplicate per point using sampling devices with porous cups (DIK-301A-A1; Daiki Rika Kogyo Co., Ltd., Saitama, Japan) every 2 weeks or every month (Fig 1). Water samples were filtered using filters with a pore size of 0.45 μm, and concentrations of K, Na, NH_4_, Mg, and Ca were measured using an ion chromatograph (Dionex ICS1500; Thermo Fisher Scientific, Waltham, MA). Plant-available K in suspended solids in water samples was not analyzed owing to the negligible contribution of this pathway. Exchangeable K discharged via surface runoff was <0.2 kg ha^−1^ crop^−1^ based on the exchangeable K content in soil (0.53 cmol kg^−1^) and suspended solid yields from a typical Japanese paddy field (0.33–0.72 t ha^−1^ crop^−1^) [6, 30]. If the yields increase by several times due to spill-over irrigation, exchangeable K discharged would be <1.0 kg ha^−1^ crop^−1^. The exchangeable K content and suspended solid yield via irrigation may be even lower.

Rice plant hills were counted, and aboveground parts were obtained from the center of the fields in the harvest season (October). Plants were separated into parts (brown rice, husk, and straw), dried for 48 h at 80°C in an oven, weighed, and ground using a mill for husk and straw. Then, 0.5 g of material was digested in 5.0 mL of HNO_3_ for 1 h at 100°C using a hot block acid digestion system (DigiPREP LS; SCP Science, Quebec, Canada) according to the methods of Kubo et al. [31]. The K concentration was determined by an atomic absorption spectrophotometer (ZA3000; Hitachi High-Tech Science Co., Tokyo, Japan).

The amount of K via pathways was calculated by multiplying the K concentration by the water volume (or plant weight) and integrating over each water sampling interval. The K budget during the observation period was determined by the following equation.

Kbudget=(F+Pr+I+S)-(R+SR+Pc), (1)

where *F* is K in applied fertilizer, *Pr* is K via precipitation, *I* is K via irrigation, *S* is K in straw returned, *R* is K in rice plant harvested, *SR* is K via surface runoff, and *Pc* is K via percolation. To quantify K leaching from plowed soil, the value for the soil solution from the lower part of plowed soil was applied to the value via percolation. The budget of rice plant-available K in plowed soil was the focus of the analysis.

## Results

### Water budget

Water was largely supplied from irrigation, especially in field A with spill-over irrigation (Table 2). Major drainage pathways differed between fields. The major pathway in field A was surface runoff, whereas that in field B was surface runoff and percolation.

**Table 2:** Water budget (mm) in fields during the cropping period.

	Inflow		Outflow		
	Irrigation	Precipitation	Surface runoff	Percolation^a^	Evapotranspiration
Field A	11066	638	10224	931	548
Field B	6303	626	3517	2814	598

^a^Mean daily percolation rates were 8 mm for field A and 24 mm for field B.

### Cation concentrations

**Fig 2:**
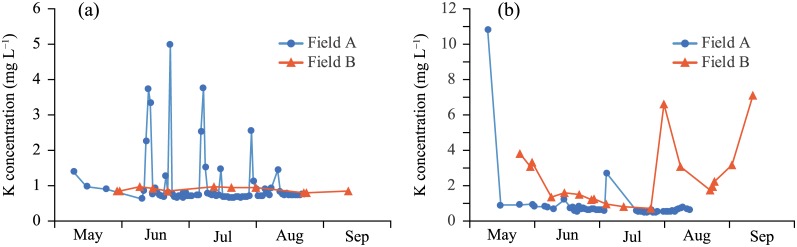
Temporal changes in K concentrations in (a) irrigation water and (b) surface runoff water during the cropping period.

The K concentrations in irrigation water were approximately 1 mg L^−1^ in both fields, with a sudden increase in field A (Fig 2a and Table 3). Other cations showed similar tendencies (S1a Fig and S1 Table). The K concentrations in precipitation were lower than those in irrigation water and less than 0.4 mg L^−1^ on average (Table 3). The K concentrations in surface runoff water were high in the early period and then decreased to the level in irrigation water (Fig 2b). The concentration increased from August only in field B. This trend was not entirely consistent with that of the other cations (S1b Fig), and the reasons for these differences are not clear.

**Table 3:** Mean K concentrations and standard deviations in pathways.

	Input			Output		
	Irrigation water (mg L^−1^)	Precipitation (mg L^−1^)	Straw (g kg^−1^)	Surface runoff water (mg L^−1^)	Soil solution^a^ (mg L^−1^)	Rice plants (g kg^−1^)
Field A	1.00 ± 0.76 (86)	0.32 ± 0.64 (10)	15 ± 0.83 (3)	0.90 ± 1.5 (50)	2.0 ± 0.67 (7)	9.0 ± 0.51 (3)
Field B	0.88 ± 0.067 (11)	0.083 ± 0.033 (5)	16 ± 1.7 (3)	2.5 ± 1.8 (18)	12 ± 6.8 (12)	9.7 ± 0.95 (3)

Sample sizes are indicated in parentheses.

^a^Values for soil solution collected from the lower layer of plowed soil.

**Fig 3:**
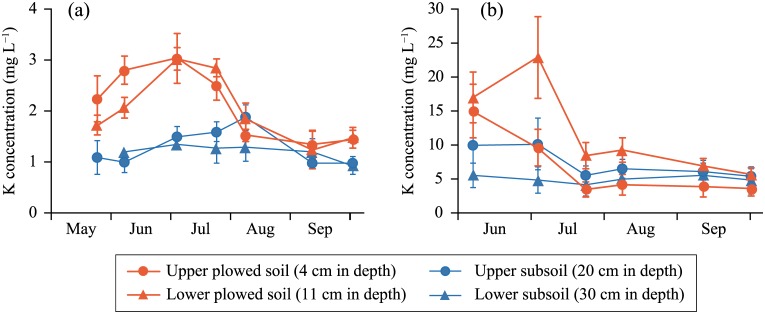
Temporal changes in K concentrations in soil solutions from (a) field A and (b) field B during the cropping period. Error bars indicate standard deviations of triplicate samples.

The K concentrations in the soil solution from plowed soil peaked at an early time point (June and July) (Fig 3). Those in the upper subsoil peaked slightly later, indicating K percolation. The concentrations in both layers then decreased to the same level. The peak was earlier in field B with greater percolation of water. The concentrations differed substantially between the fields; those in field B were an order of magnitude greater than those in field A. Although the temporal changes in the concentrations of other cations differed among fields, the concentrations were similar (S2 Fig and S1 Table).

The K concentration in rice plants did not differ between the fields (Table 3). The yields of brown rice, husk, and straw were 4.9, 1.2, and 6.0 t ha^−1^ for field A and 6.2, 1.5, and 8.2 t ha^−1^ for field B, respectively.

### K input, output, and budget

In both fields, the major pathways of K input were fertilization, irrigation, and straw return (i.e., all of the pathways excluding precipitation) (Table 4). Irrigated K accounted for 16%–48% of the total input, reflecting the large volumes (Table 2). The contribution of returned straw K was similar (40%–41%). The total input in field B was 111 kg ha^−1^ greater than that in field A, and this could be explained mainly by the difference in the fertilizer application rate.

**Table 4:** K input, output, and budget (kg ha^−1^) during the cropping period.

	Input					Output				Budget
	Fertilization	Irrigation	Precipitation	Straw return	Subtotal	Surface runoff	Percolation	Plant harvesting	Subtotal	
Field A	25	110	0.8	93	229	120	20	109	249	−20
Field B	149	56	0.7	134	340	81	392	155	629	−289

The major pathways of K output differed between the fields; these pathways were surface runoff and plant harvesting for field A and all of the pathways for field B (Table 4). Note that approximately 85% of the harvested plant K was brought back by straw return; in practice, plant harvesting was a minor pathway. Percolated K accounted for the greatest output in field B due to the high K concentration in soil solution and the high percolation rate of water. This pathway accounted for 62% of the total output and caused substantial K output in field B, which was 380 kg ha^−1^ greater than that in field A.

These sources of input and output resulted in negative K budgets in both fields. However, that in field A was nearly balanced (−20 kg ha^−1^), in contrast with the substantial negative balance in field B (−289 kg ha^−1^).

**Fig 4:**
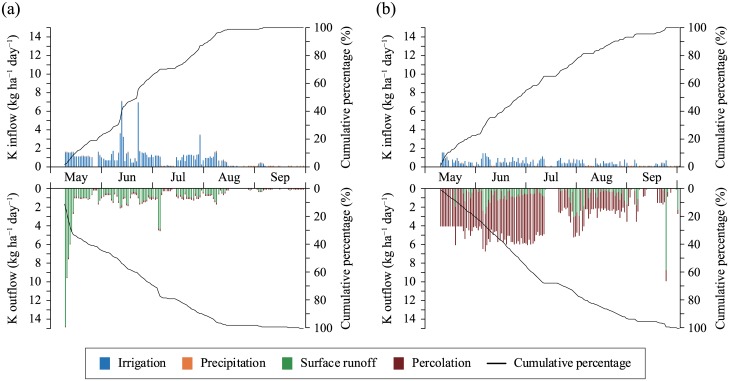
Daily K inflows and outflows and cumulative percentages in (a) field A and (b) field B during the cropping period.

As mentioned above, water flow was accompanied by the substantial input and output of K. Evaluating values on a daily basis (Fig 4), the daily K inflow did not show a remarkable trend and was less than 2 kg ha^−1^, excluding the inflow of irrigation water with a suddenly high K concentration (Fig 2). Meanwhile, 68%–77% of K outflow was discharged before the mid-summer drainage in July. In particular, a severe outflow occurred via surface runoff in the early days of cropping in field A.

## Discussion

### Factors contributing to high K output via water

Even though the total K input in field B was 111 kg ha^−1^ greater than that in field A, the K budget was highly negative (−289 kg ha^−1^) due to heavy output via percolation (Table 4). K leaching loss can be substantial in highly permeable soil with low CEC [32], as in field B (Tables 1 and 2). The plowed soil of field B had relatively high exchangeable and non-exchangeable K contents, indicating that soil rich in clay minerals strongly adsorb K. However, the CEC was less than half that of field A. This state likely resulted in severe K leaching to the soil solution despite similar concentrations of other cations, favoring K leaching in irrigation water and soil solution in both fields (S1 and S2 Figs and S1 Table). Then, the leached K was lost by the increased percolation of water. The low CEC and high permeability likely stemmed in part from the low contents of total C (i.e., organic matter) and clay (Table 1). Reducing K leaching can greatly improve the budget. Increasing CEC and/or decreasing permeability may be a practical approach.

K was also discharged in large quantities via surface runoff in the fields (Table 4). This could certainly be explained by the large irrigation volumes (11000 and 6600 mm, Table 2). A typical total inflow volume for irrigation and precipitation is around 1300–1500 mm for irrigated rice in Asia [33], and the irrigation volumes in these study fields were quite high. This irrigation system presumably favored the high surface runoff, thereby resulting in heavy K outflow. The output exceeded the input via irrigation, meaning that irrigated K was unfortunately not utilized in practice. Hoa et al. [16] have reported that the total K input via irrigation and precipitation exceeds the output in paddy fields with low volumes of surface runoff and percolation. Other factors affecting K output in these fields were similar to those in field A (heavy clay soil, CEC: 14–18 cmol_c_ kg^−1^, K application rates: 13–75 kg ha^−1^ crop^−1^, exchangeable K contents: 0.25–0.46 cmol K kg^−1^). Thus, irrigation water-saving could contribute to the utilization of irrigated K. Decreasing permeability in field B mentioned above would increase the volume of surface runoff water and K outflow accompanied with it, and irrigation water-saving should be combined. Because 68%–77% of the K outflow occurred before mid-summer drainage (Fig 4), these approaches should be implemented in the early period. Water-saving may be limited to a shorter time period in field A, where severe outflow via surface runoff subsided in the early days of cropping. A shortfall of K in the budgets may also be addressed after leaching and runoff conditions improve.

The activity concentration of ^137^Cs was less than 10 Bq kg^−1^ for brown rice harvested from the fields (unpublished data) and met the Japanese standard of 100 Bq kg^−1^. Accordingly, radiocesium uptake has been controlled by countermeasures aimed at enhancing exchangeable K. However, exchangeable (and non-exchangeable) K contents would decline at this rate or after the termination of countermeasures, especially in paddy fields similar to field B. Deeper discussions of K maintenance are needed to prepare for such a case.

## Conclusions

This study aimed to clarify the major K input and output pathways that determine the budgets in two paddy fields in which countermeasures were implemented against radiocesium uptake in Fukushima. The findings of this study provide basic information on K maintenance. We revealed that water outflow is an important determinant of the budget in fields with high-volume irrigation and percolation. Negative budgets may be improved by controlling the volume, increasing CEC, and decreasing permeability. However, the effects of these factors should be verified by column, pot, or field experiments. Appropriate K application rates should be decided following these improvements. Radiocesium derived from the Fukushima nuclear accident was deposited over a vast area. Naturally, soil properties and water conditions, including irrigation, drainage, and K concentration, exhibited high variation. Further studies considering a wide range of conditions are necessary to develop a plan for K maintenance.

## Supporting information

S1 Fig Temporal changes in ion concentrations of Na, NH_4_, Mg, and Ca in (a) irrigation water and (b) surface runoff water during the cropping period. (EPS)

S2 Fig Temporal changes in ion concentrations of Na, NH_4_, Mg, and Ca in soil solution at (a) field A and (b) field B during the cropping period. Error bars indicate standard deviations of triplicate samples. (EPS)

S1 Table Mean ion concentrations and standard deviations (mg L^−1^) of Na, NH_4_, Mg, and Ca in water pathways. (DOCX)
