# Supplementary material for: Input and output pathways determining potassium budgets in two paddy fields subjected to countermeasures against radiocesium in Fukushima, Japan
Source: PLoS One. 2020 Apr 24;15(4):e0232139. doi: 10.1371/journal.pone.0232139 (PMC7182197; doi:10.1371/journal.pone.0232139)
Supplement: S1 Table — (DOCX) [file pone.0232139.s003.docx]

**Table S1 Mean ion concentrations and standard deviations (mg L^−1^) of Na, NH_4_, Mg, and Ca in water pathways.**

|  |  | Sample size | Na | NH_4_ | Mg | Ca |
| --- | --- | --- | --- | --- | --- | --- |
| Irrigation water | Field A | 86 | 5.3 ± 0.59 | 0.12 ± 0.50 | 2.5 ± 3.7 | 7.6 ± 7.1 |
|  | Field B | 11 | 5.2 ± 0.42 | 0.0090 ± 0.0065 | 1.1 ± 0.13 | 4.9 ± 0.62 |
| Precipitation | Field A | 10 | 1.5 ± 1.5 | 0.73 ± 1.2 | 0.22 ± 0.22 | 0.42 ± 0.54 |
|  | Field B | 5 | 1.4 ± 1.1 | 0.43 ± 0.35 | 0.16 ± 0.092 | 0.75 ± 0.53 |
| Surface runoff water | Field A | 50 | 5.1 ± 0.45 | 0.11 ± 0.34 | 1.4 ± 0.41 | 1.4 ± 0.41 |
|  | Field B | 18 | 4.8 ± 0.99 | 0.21 ± 0.65 | 0.94 ± 0.32 | 3.5 ± 1.5 |
| Soil solution^a^ | Field A | 7 | 8.2 ± 3.0 | 0.29 ± 0.37 | 5.7 ± 3.7 | 19 ± 11 |
|  | Field B | 12 | 5.6 ± 1.2 | 0.63 ± 0.94 | 6.3 ± 3.1 | 13 ± 6.0 |

^a^Values in soil water collected from the lower layer of plowed soil.
